# SMART COVID Navigator, a Clinical Decision Support Tool for COVID-19 Treatment: Design and Development Study

**DOI:** 10.2196/29279

**Published:** 2022-02-18

**Authors:** Varun Suraj, Catherine Del Vecchio Fitz, Laura B Kleiman, Suresh K Bhavnani, Chinmay Jani, Surbhi Shah, Rana R McKay, Jeremy Warner, Gil Alterovitz

**Affiliations:** 1 Biomedical Cybernetics Laboratory Brigham and Women's Hospital Boston, MA United States; 2 Reboot Rx Boston, MA United States; 3 Preventive Medicine and Population Health Institute for Translational Sciences University of Texas Medical Branch University of Texas Health Science Center in Houston Houston, TX United States; 4 Department of Internal Medicine Mount Auburn Hospital Harvard Medical School Cambridge, MA United States; 5 Hematology, Oncology and Bone Marrow Transplantation Mayo Clinic Phoenix, AZ United States; 6 Department of Medicine and Urology University of California San Diego, CA United States; 7 Medicine and Biomedical Informatics Vanderbilt University Nashville, TN United States; 8 Biomedical Cybernetics Laboratory Brigham and Women’s Hospital Harvard Medical School Boston, MA United States

**Keywords:** COVID-19, clinical decision support, precision medicine, web application, FHIR

## Abstract

**Background:**

COVID-19 caused by SARS-CoV-2 has infected 219 million individuals at the time of writing of this paper. A large volume of research findings from observational studies about disease interactions with COVID-19 is being produced almost daily, making it difficult for physicians to keep track of the latest information on COVID-19’s effect on patients with certain pre-existing conditions.

**Objective:**

In this paper, we describe the creation of a clinical decision support tool, the SMART COVID Navigator, a web application to assist clinicians in treating patients with COVID-19. Our application allows clinicians to access a patient’s electronic health records and identify disease interactions from a large set of observational research studies that affect the severity and fatality due to COVID-19.

**Methods:**

The SMART COVID Navigator takes a 2-pronged approach to clinical decision support. The first part is a connection to electronic health record servers, allowing the application to access a patient’s medical conditions. The second is accessing data sets with information from various observational studies to determine the latest research findings about COVID-19 outcomes for patients with certain medical conditions. By connecting these 2 data sources, users can see how a patient’s medical history will affect their COVID-19 outcomes.

**Results:**

The SMART COVID Navigator aggregates patient health information from multiple Fast Healthcare Interoperability Resources–enabled electronic health record systems. This allows physicians to see a comprehensive view of patient health records. The application accesses 2 data sets of over 1100 research studies to provide information on the fatality and severity of COVID-19 for several pre-existing conditions. We also analyzed the results of the collected studies to determine which medical conditions result in an increased chance of severity and fatality of COVID-19 progression. We found that certain conditions result in a higher likelihood of severity and fatality probabilities. We also analyze various cancer tissues and find that the probabilities for fatality vary greatly depending on the tissue being examined.

**Conclusions:**

The SMART COVID Navigator allows physicians to predict the fatality and severity of COVID-19 progression given a particular patient’s medical conditions. This can allow physicians to determine how aggressively to treat patients infected with COVID-19 and to prioritize different patients for treatment considering their prior medical conditions.

## Introduction

Precision medicine, as defined by the National Institutes of Health’s Precision Medicine Initiative, is “an emerging approach to disease treatment and prevention that takes into account individual variability in genes, environment, and lifestyle for each person” [1]. Recent increases in the availability of electronic patient data have facilitated the development of precision medicine. For instance, electronic health records (EHRs) store all information collected in hospitals, such as blood tests, radiographs, diagnostic tests, and any biographical information about a patient, such as their age, weight, or height [2]. This approach to medical treatment could prove to be useful in dealing with the COVID-19 caused by SARS-CoV-2, which has been ravaging the United States and the world [3,4]. Currently, a vast amount of research is being conducted to understand how a patient’s underlying health conditions interact with the progression of the virus infection. For example, certain conditions such as diabetes and heart disease have been found to raise the severity and fatality rates of patients who have become infected with the virus [5]. Applying the tools of precision medicine can help doctors customize treatment for patients affected by COVID-19 based on the patient’s underlying conditions. Attempts at clinical decision support systems for COVID-19 have been made using information on risk factors and biomarker measurements [6,7].

With the rapid spread of COVID-19 and the scarcity of physician resources and time, there is an immediate need for a clinical decision support system that provides patient and disease interaction information to clinicians to allow them to practice precision medicine. In this paper, we describe the creation of the SMART COVID Navigator, a web-based application designed to assist clinicians to relate patient risk factors to the growing amount of research that identifies how various underlying patient conditions affect the progression of COVID-19. This application identifies patient risk factors based on integrating comprehensive data available through multiple EHRs. The application then allows clinicians to quickly access a large set of research studies based on their patient’s medical history in an easy-to-access format, helping promote updated information on COVID-19 and its risks for a diverse community. This tool will simplify a clinician’s search for relevant research and findings and support clinical treatment.

The SMART COVID Navigator connects patient information from multiple EHR servers to 2 databases of COVID-19 research studies. This will allow clinicians to access data-driven research based on a particular patient’s risk factors. The SMART COVID Navigator builds on the framework developed by the SMART Cancer Navigator, which offers clinical decision support by connecting patient EHR information to cancerous gene variants [8,9]. The Navigator is a further step for creating Fast Healthcare Interoperability Resources (FHIR) protocol–based tools to support personalized medicine [10]. This web-based application was built by researchers at the Biomedical Cybernetics Laboratory in Harvard Medical School. It was created using an Angular and Bootstrap front-end framework. The code is available at [11].

## Methods

### Accessing Multiple FHIR Servers

The SMART COVID Navigator allows the user to log into 2 EHRs: the Veterans Affairs (VA) and the Center for Medicare and Medicaid Services (CMS). There are significant advantages to the application being able to log into multiple EHR servers [12]. Doctors viewing their patient’s medical information through the application have the ability to discern any discrepancies between the data sources. Similarly, doctors and patients can be sure that they are receiving the most up-to-date information regarding their health records, as any information not captured in one of the EHRs would most likely be shown in the other. The VA and CMS servers follow the FHIR standard [13]. The FHIR platform allows for the interoperability of the navigator with other EHRs that follow the FHIR protocol. The application is registered with the relevant EHRs. The system can be expanded to access additional health records, provided their application programming interfaces (APIs) follow the FHIR standard. The EHRs provide the application with a client ID and a client secret, which is used for authentication purposes. The following section describes the login process.

**Figure 1:**
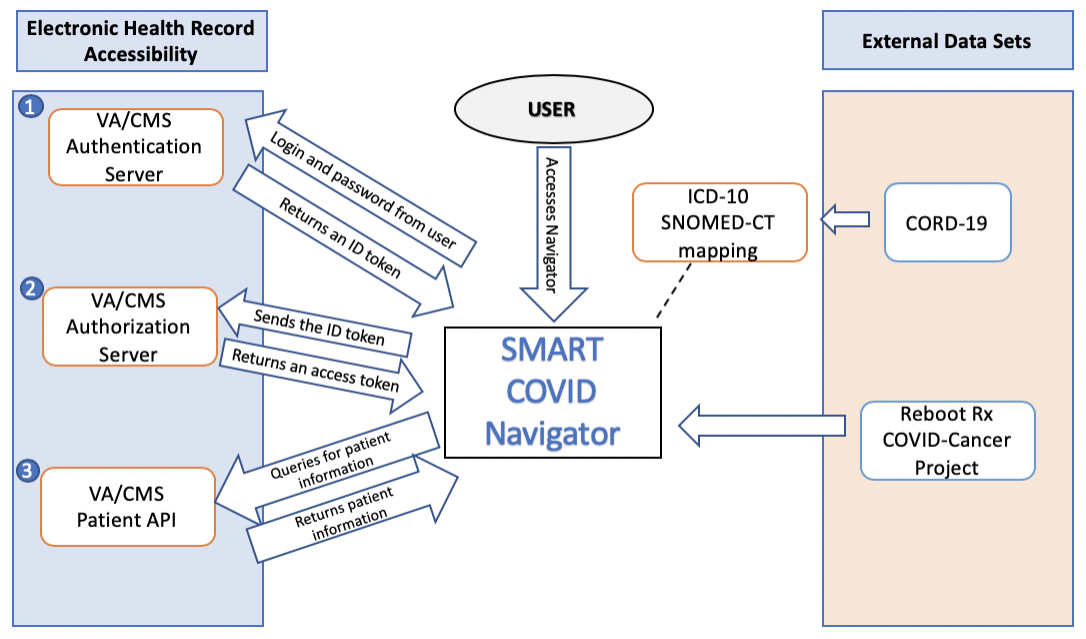
Architecture diagram of the SMART COVID Navigator. API: application programming interface; CMS: Center for Medicare and Medicaid Services; CORD-19: COVID-19 Open Research Dataset; ICD-10: International Classification of Diseases-tenth version; SNOMED-CT: SNOMED Clinical Terms; VA: Veterans Affairs.

To log into the 2 API-enabled EHR systems, the application implements the OAuth2 [14] and OpenID Connect [15] standards to achieve secure authentication and authorization. The authentication process involves a user entering their login credentials, while the authorization process requires a request to the EHR’s server to obtain an access token. Upon receiving the access token, the application is authorized to retrieve relevant patient information. The left side of Figure 1 depicts the system architecture for the EHR access process. The login process begins when the user clicks one of the login buttons. The user is redirected to the login portal for the EHR of their choice to enter their login credentials. The OAuth2 process redirects the user back to the application with an ID token in the URL (step 1 in Figure 1). With this ID token, the application then requests an access token from the relevant API to obtain access to the patient’s medical information. The application accomplishes this second step by sending the ID token along with additional information such as the application’s client ID and client secret back to the EHR server through an HTML POST request. The server then returns an access token, which the application can use to gain access to any part of the patient’s profile (step 2 in Figure 1). The access token is saved to the local storage of the app; thus, if the user refreshes the application or attempts to log into another EHR, they will still retain access to the first EHR. This 2-step process ensures better security of sensitive medical data. From the EHRs, the following information is retrieved: (1) the patient’s name, (2) their location zip code, (3) their date of birth (which is used to calculate their age), and (4) a list of medical conditions associated with the patient along with a numerical code presenting the condition (step 3 in Figure 1).

**Figure 2:**
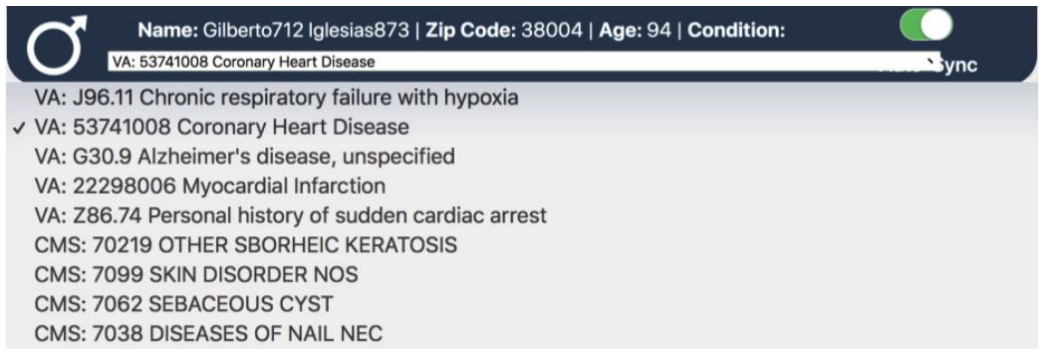
The disease condition list for a Veterans Affairs test patient. This dropdown appears from the patient header.

The EHR server results are visually displayed at the top of the screen. This menu appears when a user logs into either one of the servers. It displays demographic information such as the patient’s name, age, and current zip code. It also shows a list of medical conditions retrieved from the patient’s profile with the list of conditions appearing in a dropdown menu (Figure 2). If the user is logged into both the VA and CMS servers, then the application retrieves demographic information from the VA server, while the condition list is a combined list from both EHRs. Please note that the patient information displayed in Figure 2 and in other figures below are based on information taken from VA- and CMS-simulated patient data; since these data are not from a real patient, there are no Health Insurance Portability and Accountability Act concerns. With these patient conditions now available in the Navigator, the next step is to connect these conditions to risk factors shown to affect COVID-19 progression.

### Accessing Artificial Intelligence–Powered Knowledge Base From the COVID-19 Open Research Dataset

**Figure 3:**
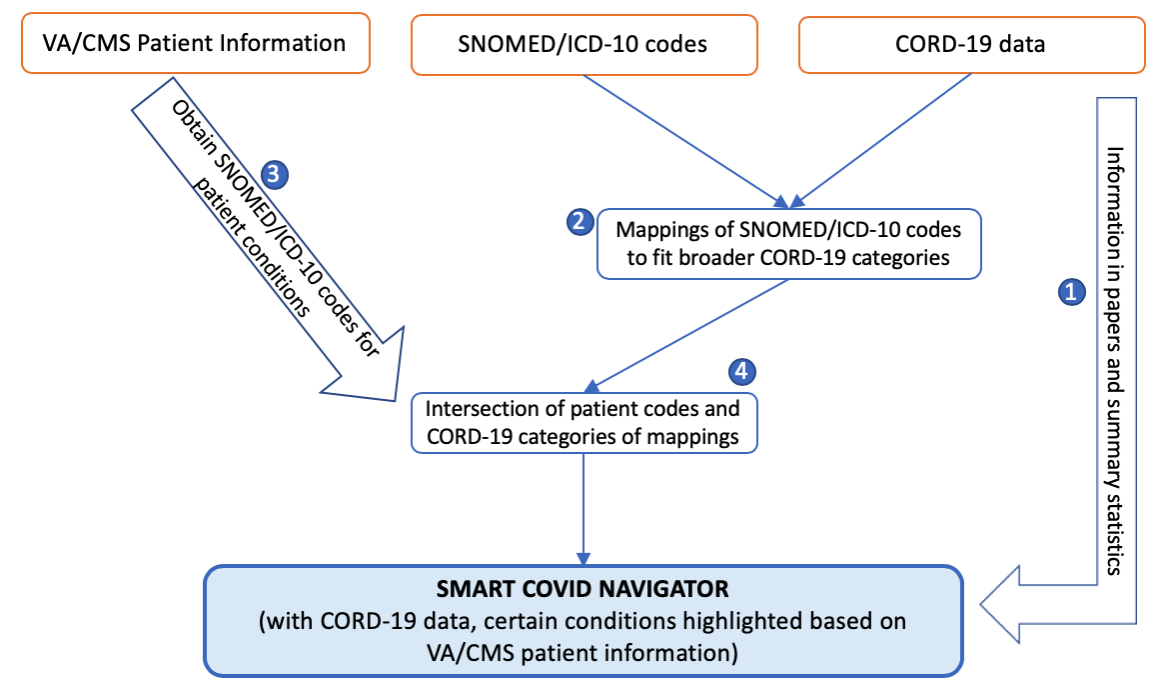
Architecture diagram of the interactions between the Veterans Affairs and Center for Medicare and Medicaid Services electronic health record systems, SNOMED/International Classification of Diseases-tenth version codes, and the COVID-19 Open Research Dataset. CMS: Center for Medicare and Medicaid Services; CORD-19: COVID-19 Open Research Dataset; ICD-10: International Classification of Diseases-tenth version; SNOMED-CT: SNOMED Clinical Terms; VA: Veterans Affairs.

The SMART COVID Navigator collects risk factor information from 2 external data sets, as shown on the right side of Figure 1 and relates it to patient medical conditions. The first data set is the COVID-19 Open Research Dataset (CORD-19), created by the White House and a set of leading research groups such as the Georgetown University’s Center for Security and Emerging Technology and the National Library of Medicine, National Institutes of Health [16]. This resource currently contains over 200,000 scholarly papers related to COVID-19. Researchers apply artificial intelligence techniques to create this knowledge base. CORD-19 sources papers from the World Health Organization, PubMed Central, bioRxiv, and medRxiv. These can be in the form of physical printouts, PDF files, or XML files. The papers and preprints are then collected by Semantic Scholar [17], and the resulting metadata are harmonized and deduplicated. The full text of the papers is then extracted [18]. This knowledge base is stored on Kaggle, a web-based community of data scientists and machine learning practitioners [19]. The Navigator utilizes the “risk factors” section of CORD-19 to support clinical assessment of how a patient’s medical conditions affect their chances of having a severe or fatal COVID-19 infection (step 1 in Figure 3).

The CORD-19 data are publicly available and are stored in CSV files. Each file tracks studies relating to a specific condition (eg, hypertension, heart disease) that is being tracked. Each row in the CSV file represents a study. The study name, link, date, and significance of the severity and fatality of the patients are all provided. Textbox 1 provides a list of the risk factors provided in CORD-19. As shown in Textbox 1, the CORD-19 data set consists of 28 risk factors—22 of which are disease conditions, while the other 6 are patient biographical characteristics. The comprehensive clinical terminology coding systems, SNOMED Clinical Terms used by the VA [20] and the International Classification of Diseases-tenth version (ICD-10) used by CMS [21], consist of thousands of more specific disease identifiers than the broader disease categories tracked by CORD-19. We mapped the identifiers in the SNOMED Clinical Terms and ICD-10 code sets to the CORD-19 risk factors to be able to match patient conditions to disease categories tracked by CORD-19 (step 2 in Figure 3). This mapping was accomplished through word matching. For each of the 28 risk factors present in CORD-19, the name of the risk factor or a part of the name was matched with every instance of that name occurring in the SNOMED Clinical Terms and ICD-10 code sets by using a Python script created by the authors. For instance, the risk factor “diabetes” links to any disease classification in the code sets that has the word “diabetes” or variations such as “diabetic.” Because the disease identifiers in the code sets are more specific than the broad risk factors tracked by CORD-19, many risk factors align with hundreds of more specific disease names from the code sets. Since the mapping involved matching words or parts of words using Python code (and was not done manually), we believe there is unlikely to be incorrect mapping. For example, we searched for the string “diabet,” which would capture both “diabetes” and “diabetic.” Nevertheless, it is possible that some conditions in the 2 code sets might not have been captured despite our attempts at careful mapping.

**Risk factors tracked by the COVID-19 Open Research Dataset.**

**Risk factors**

- Age
- Endocrine diseases
- Asthma
- Ethnicity: Hispanic versus non-Hispanic
- Autoimmune disorders
- Heart disease
- Chronic obstructive pulmonary disease 
- Heart failure
- Cancer
- Hypertension
- Cardiovascular and cerebrovascular disease
- Immune system disorders
- Cerebrovascular disease
- Male gender
- Chronic digestive disorders
- Neurological disorders
- Chronic kidney disease
- Overweight or obese
- Chronic liver disease
- Race: Asian versus White
- Chronic respiratory diseases
- Race: Black versus White
- Dementia
- Race: Other versus White
- Diabetes
- Respiratory system diseases
- Drinking
- Smoking status

**Figure 4:**
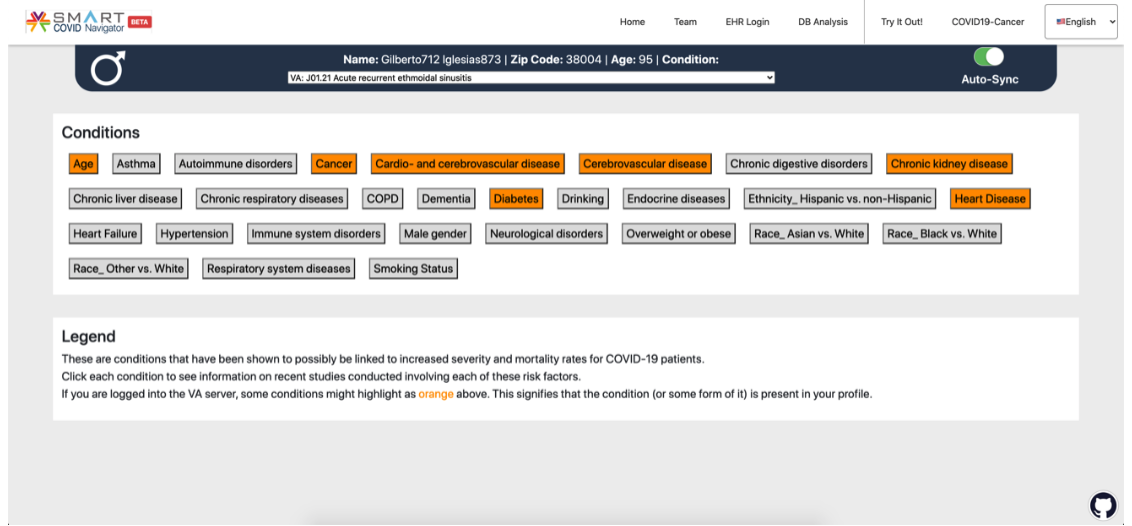
A view of the application logged into a test Veterans Affairs profile. A button appears for each condition that is being tracked. Based on the conditions in the patient’s profile, certain risk factors on the screen are highlighted in orange.

**Figure 5:**
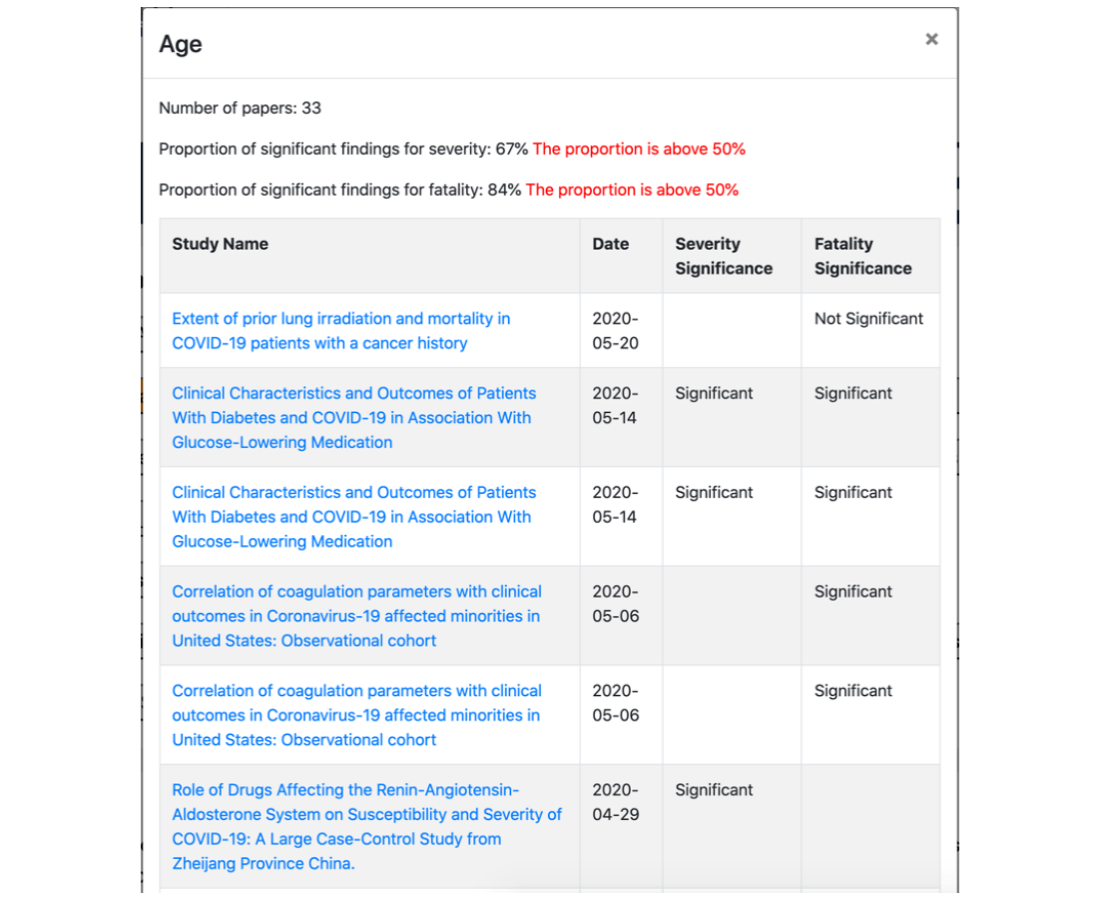
Presentation of information from the COVID-19 Open Research Dataset relating to a particular risk factor (in this case, the example is for the “age” risk factor).

Visually in the Navigator, the information from CORD-19 regarding studies related to COVID-19 risk factors appears as a menu of conditions (Figure 4). When the user clicks a particular condition, a pop-up screen is displayed, providing all the information collected about studies relevant to that condition (Figure 5). This will allow a user to view the studies and use the findings for clinical decision support. The study name (with a hyperlink to the full study), date, and the significance of the severity and fatality statistics are shown for each study. At the top of the pop-up, an overview of the studies associated with the chosen condition is displayed. The information included are the total number of studies for that condition, the percentage of studies that found the selected condition to cause a significant change in the severity of COVID-19 progression (out of the studies that measure for severity), and the percentage of studies that found a significant change in fatality due to COVID-19. We alert the user if the proportion of papers finding a significant result is greater than 50%. If a particular risk factor is found to be associated with the severity or fatality due to COVID-19 infection in a high proportion of observational studies, then physicians should pay more attention to patients with that risk factor. However, individual physicians should make their own decisions based on the information in the SMART COVID Navigator and the severity of their patients’ health conditions.

The patient’s biographical and health information retrieved from the EHRs can be used to better guide clinicians on the health conditions they should be concerned about in relation to COVID-19. The Navigator uses the previously described mappings from SNOMED Clinical Terms and ICD-10 identifiers to the CORD-19 risk factors; wherever there is a match between the code in a patient’s EHR condition list (step 3 of Figure 3) and those associated with each CORD-19 risk factor, the condition highlights orange (step 4 of Figure 3), thereby alerting the user to presence of the condition of concern (Figure 4). If the patient is older than 60 years, then the “age” condition is highlighted as well. The clinician should click on any highlighted conditions to access further information from the CORD-19 database, as this information is likely to be relevant for the patient in question.

### COVID-19 in Patients With Cancer

Cancer has been shown to be a risk factor for COVID-19, and the different types of cancers affect COVID-19 progression differently [22]; therefore, it is important for physicians to understand disease progression if patients with cancer become infected with COVID-19. To help with this, the SMART COVID Navigator accesses Reboot Rx’s Reboot: COVID-Cancer Project data sets in order to display information regarding the impact of COVID-19 and its treatment on patients with cancer [23]. The Reboot: COVID-Cancer Project identifies relevant published clinical studies and extracts and aggregates the data from those studies into 2 data sets. The first data set examines COVID-19 disease progression for patients with cancer. The second data set examines what effect drugs currently being tested for COVID-19 treatment could have on cancer (independent of their effect on COVID-19). The data sets are publicly available on the Reboot: COVID-Cancer Project website via interaction dashboards and can be requested in the form of Excel files. This feature gives an organized tool for clinicians to better understand COVID-19 outcomes for different types of cancer. Textbox 2 shows each of the tissue types that are examined in the Reboot: COVID-Cancer Project data sets (the numbers in parentheses indicate which data sets the tissue type is in). As shown in Textbox 2, the data sets consist of 30 tissue types. Tissue types were chosen to distinguish the cancers instead of cancer types, as the number of tissue types was more manageable and therefore would be easier to access for the user of the application.

**Figure 6:**
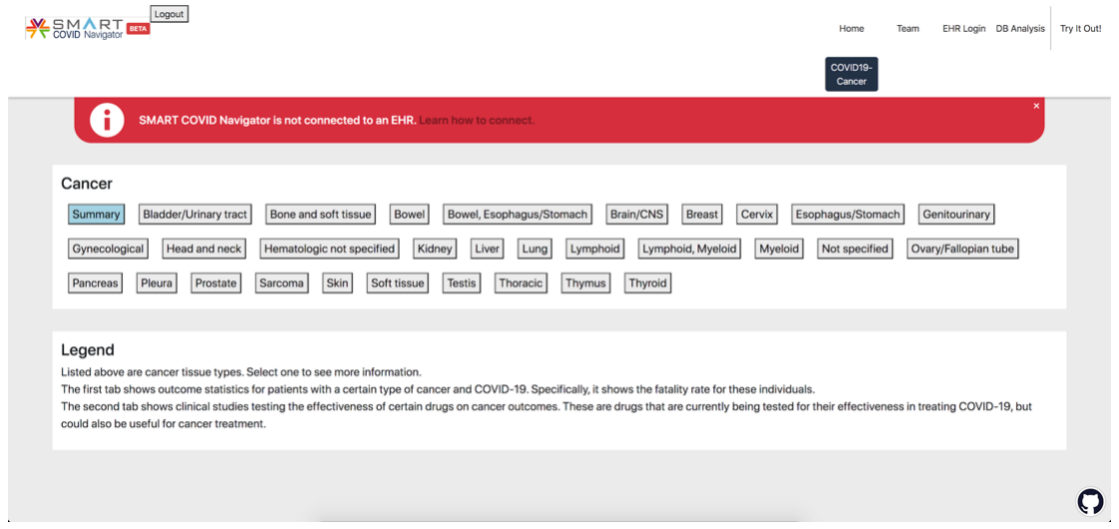
Standard view of the COVID-19 cancer tab of the COVID Navigator. The “Summary” button is highlighted in blue to distinguish it from the other buttons that represent tissue types.

**Figure 7:**
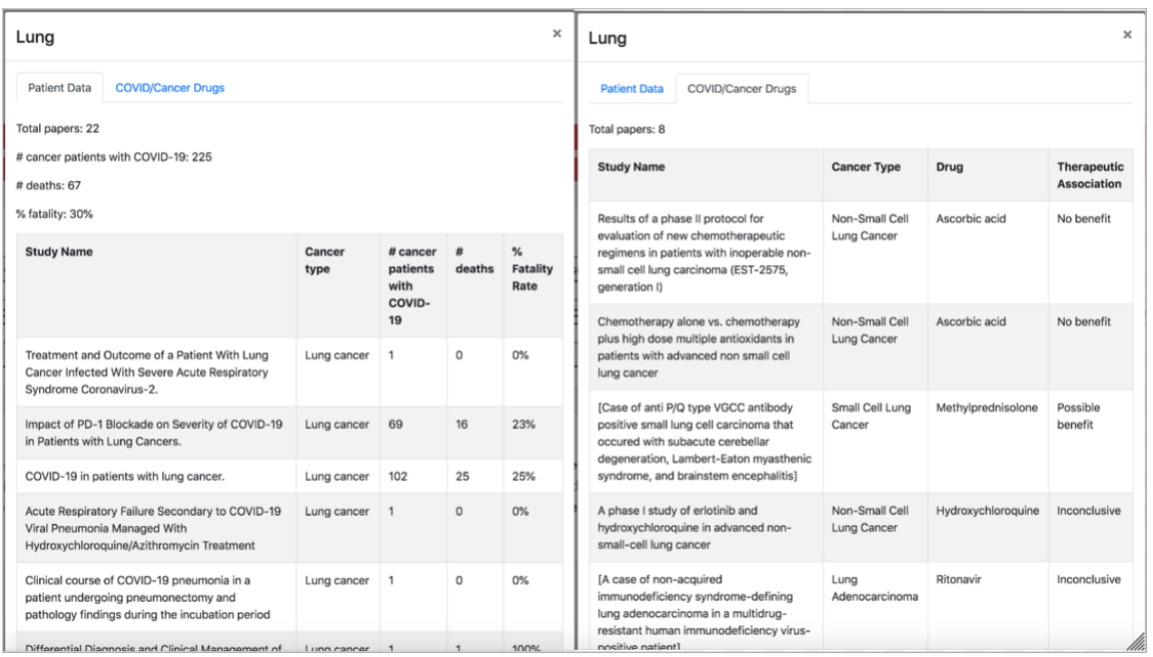
When a tissue is clicked, a pop-up appears with 2 tabs (this figure shows the pop-up for lung tissue). The first tab (on the left) tracks patient outcomes for patients with lung cancer who contract COVID-19. The second tab (on the right) tracks drugs being tested for COVID-19 that could have an impact on cancer of the selected tissue.

**Figure 8:**
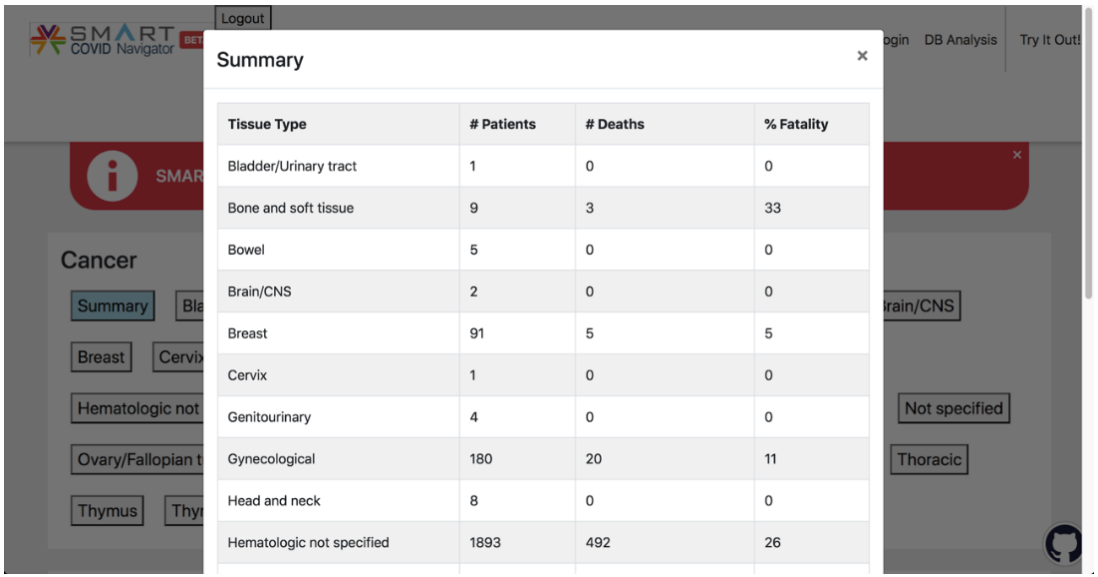
When the blue “Summary” button is clicked, this pop-up appears, which displays the patient outcome summary statistics for each tissue type in a concise manner.

When a user navigates to the “COVID19-Cancer” tab of the SMART COVID Navigator, they will see a menu of tissue types, similar to how the CORD-19 risk factors are displayed, as well as another button that displays the summary information for all of the tissues in 1 pop-up, colored blue to distinguish it from the other buttons representing tissue types (Figure 6). When one of the buttons is clicked, a pop-up appears with 2 tabs. The first tab displays information retrieved from data set 1 regarding patient outcomes, and the second tab displays information from data set 2 about COVID-19 drugs that could be useful for cancer treatment (Figure 7). When the summary tab is clicked, information retrieved from data set 1 about all tissue types in that data set is displayed, so that clinicians can easily see all the patient outcome statistics on 1 central page (Figure 8). Please note that the use of data set 1 in the SMART COVID Navigator is to display patient outcomes for the treatment of COVID-19 in patients with cancer. This use is similar to how we used the CORD-19 data set. The display of data set 2 is for a different purpose—to inform physicians treating cancer that how drugs that are being used for COVID-19 treatment have an application in cancer treatment.

**Tissue types tracked by the Reboot: COVID-Cancer Project data sets. The numbers in parentheses indicate which data sets the tissue type is in.**

**Tissue types**

- Bladder/unitary tract (1,2)
- Head and neck (1,2)
- Pancreas (2)
- Bone and soft tissue (1)
- Hematologic not specified (1)
- Pleura (1)
- Bowel (1,2)
- Kidney (1,2)
- Prostate (1,2)
- Bowel, esophagus/stomach (2)
- Liver (1,2)
- Sarcoma (2)
- Brain/central nervous system (1,2)
- Lung (1,2)
- Skin (1,2)
- Breast (1,2)
- Lymphoid (1,2)
- Soft tissue (2)
- Cervix (1)
- Lymphoid, myeloid (2)
- Testis (2)
- Esophagus/stomach (2)
- Myeloid (1,2)
- Thoracic (1)
- Genitourinary (1)
- Not specified (1,2)
- Thymus (1,2)
- Gynecological (1)
- Ovary/fallopian tube (1,2)
- Thyroid (2)

### Data Governance

There are 2 sources of data used in the application. The first is patient data from the VA and CMS, which are accessed when a patient logs into their accounts in these EHRs. The SMART COVID Navigator does not store any patient data. These data are accessed by the patient or physician under authorization by the patient. These data are used to identify patient risk factors when the physicians/patients are using the application. When they are done using the application and log out, the patient data are deleted from the local machine, which is being used to access the app. At no point is patient data stored in the main server running the application. The second source of data is from the publicly available CORD-19 and Reboot: COVID-Cancer Project data sets for use in the application. Given these are publicly available repositories of research papers, we do not implement any specific data governance with respect to this information.

## Results

### Knowledge Base Analysis

In addition to the development of the SMART COVID Navigator, we performed analyses on the research studies in the CORD-19 and the Reboot: COVID-Cancer Project knowledge bases. Given the large number of studies both in the CORD-19 knowledge base and the COVID-Cancer data sets, it would be difficult for a clinician to quickly assess if a particular patient’s disease condition or cancer type interacts significantly with COVID-19. For the CORD-19 knowledge base, we explored whether on average, the studies found significant results for each of the 28 conditions examined, and for the Reboot: COVID-Cancer Project data sets, we explored the likelihood of mortality for a particular cancer type based on the aggregate data collected from the data sets.

### CORD-19 Knowledge Base Analysis

First, we discuss the results from the CORD-19 knowledge base relating to risk factors. We found that out of the 816 studies examined in the risk factors section, 355 studies (43.5%) studied only how a particular disease condition affected the severity of COVID-19 progression, 328 studies (40.2%) examined only how the disease condition affected fatality, 89 studies (10.9%) examined both severity and fatality, and 44 studies (5.4%) had no entry for severity nor fatality. We examined each of the 28 individual risk conditions to identify which of them have been found more frequently to be a significant factor in COVID-19 severity and fatality rates. These data are displayed in Tables 1 and 2. Table 1 presents the data for each condition regarding severity, and Table 2 presents the data regarding fatality.

**Table 1:** Severity statistics for all risk factors.

Risk factor	Total papers (N)	Studies on COVID-19 severity (n)	Studies showing significant severity, n (%)^a^
Age	33	15	10 (67)
Asthma	6	3	0 (0)
Autoimmune disorders	3	1	0 (0)
Cancer	26	16	8 (50)
Cardiovascular and cerebrovascular disease	10	7	4 (57)
Cerebrovascular disease	20	8	6 (75)
Chronic digestive disorders	5	2	0 (0)
Chronic kidney disease	38	19	10 (53)
Chronic liver disease	12	6	1 (17)
Chronic respiratory diseases	29	12	6 (50)
Chronic obstructive pulmonary disease	41	23	18 (78)
Dementia	5	2	1 (50)
Diabetes	100	57	33 (58)
Drinking	1	1	0 (0)
Endocrine diseases	4	4	2 (50)
Ethnicity: Hispanic versus non-Hispanic	15	6	0 (0)
Heart disease	98	54	42 (78)
Heart failure	17	3	1 (33)
Hypertension	100	60	35 (58)
Immune system disorders	6	4	3 (75)
Male gender	100	59	29 (49)
Neurological disorders	6	3	1 (33)
Overweight or obese	43	28	18 (64)
Race: Asian versus White	5	2	0 (0)
Race: Black versus White	21	7	2 (29)
Race: Other versus White	9	3	0 (0)
Respiratory system diseases	7	6	5 (83)
Smoking status	56	33	16 (48)

^a^The proportion of studies showing significant severity was calculated from the number of studies showing severity of COVID-19 in people with the particular risk factor.

**Table 2:** Fatality statistics for all risk factors.

Risk factor	Total papers (N)	Studies on fatality (n)	Studies with significant fatality value, n (%)^a^
Age	33	19	16 (84)
Asthma	6	4	2 (50)
Autoimmune disorders	3	3	2 (67)
Cancer	26	13	6 (46)
Cardiovascular and cerebrovascular disease	10	3	2 (67)
Cerebrovascular disease	20	14	10 (71)
Chronic digestive disorders	5	2	0 (0)
Chronic kidney disease	38	23	14 (61)
Chronic liver disease	12	5	3 (60)
Chronic respiratory diseases	29	19	13 (68)
Chronic obstructive pulmonary disease	41	19	8 (42)
Dementia	5	4	4 (100)
Diabetes	100	46	26 (57)
Drinking	1	0	N/A^b^
Endocrine diseases	4	0	N/A
Ethnicity: Hispanic versus non-Hispanic	15	11	6 (55)
Heart disease	98	51	43 (84)
Heart failure	17	14	9 (64)
Hypertension	100	43	21 (49)
Immune system disorders	6	4	4 (100)
Male gender	100	39	20 (51)
Neurological disorders	6	3	3 (100)
Overweight or obese	43	20	12 (60)
Race: Asian versus White	5	4	3 (75)
Race: Black versus White	21	17	7 (41)
Race: Other versus White	9	9	3 (33)
Respiratory system diseases	7	2	2 (100)
Smoking status	56	26	5 (19)

^a^The proportion of studies showing significant fatality was calculated from the number of studies showing fatality of COVID-19 in people with the particular risk factor.

^b^N/A: not applicable.

Table 1 documents severity statistics; specifically, the number and proportion of studies that find if a given condition results in a statistically significant change in the likelihood of a patient having a severe COVID-19 infection. For example, out of the 98 studies on heart disease, 54 of them study severity and 42 (78%) found a significant result for severity. However, chronic liver disease seems to not have a significant impact on COVID-19 severity, as out of the 12 papers that study this condition, 6 study severity and only 1 study (17%) found a significant correlation. This application alerts users if more than 50% of papers report a significant finding.

Table 2 is similar to Table 1, except that instead of documenting findings about COVID-19 severity, it documents statistics regarding fatality. We can again look at some examples of medical conditions. As in the case of severity of COVID-19 progression, heart disease is a useful predictor of COVID-19 fatality; out of the 51 papers that measure fatality statistics for the condition, 43 of them (84%) found a significant correlation. Being a smoker, however, does not seem to have the same high degree of correlation; only 5 (19%) out of the 26 papers that measure fatality statistics for smoking status found a significant result. Similarly to that for severity, the application alerts users if more than 50% of papers report a significant finding for fatality.

There are conditions for which the papers tracked by CORD-19 do not offer a definitive answer. Cancer is one of these conditions; 50% (8/16) of studies found a significant finding for severity, while 46% (6/13) found a significant finding for fatality. A possible explanation for this inconclusive result is the existence of various types of cancers in different tissues of the body, resulting in a mixed result when grouping various cancer types together. For such cases, clinicians will need to access additional information from other papers. As discussed before, we display the severity and fatality percentage summary statistics in the application for each condition so that clinicians can obtain a quick overview of the significance of that condition without having to review each study in detail. However, we caution that clinicians might want to review the studies in greater detail depending on the medical condition of the concerned patient.

### Reboot: COVID-Cancer Project Knowledge Base Analysis

In addition to the CORD-19 analysis, we conducted an analysis based on the papers in the Reboot: COVID-Cancer Project data set relating to patient outcomes. This data set stores the number of patients with a specific type of cancer and with COVID-19, the number of those patients who died, and the percentage fatality found. The Navigator aggregates these data to offer the user summary statistics for each of the tissue types. Table 3 documents the aggregate patient outcomes for each cancerous tissue, compiled by adding the number of patients with COVID-19 and number of deaths reported in all the papers relating to the given tissue type. Some cancer tissue types in the table can be seen to be associated with a relatively high fatality rate. For instance, out of the 200 patients with thoracic cancer, 66 died (33%) due to COVID-19. Similarly, 152 out of the 504 patients with lymphoid cancer died (30.1%) and 67 of the 225 patients with lung cancer died (29.7%), indicating that cancers of these tissue types generally result in a higher fatality rate for COVID-19. However, only 5 out of the 91 patients with breast cancer died (6%), indicating that breast cancer is associated to a lesser degree with COVID-19 fatality than some other cancer tissue types.

For some tissue types, the data available through the Reboot: COVID-Cancer Project do not contain an adequate number of patients to support clinical decisions. For instance, based on the fatality rate of 33% (3/9), it would seem that bone and soft tissue cancer is associated with a high risk of COVID-19 fatality. However, the data contain only 9 patients with bone and soft tissue cancer; therefore, more information is needed to make clinical decisions for patients with COVID-19 and bone and soft tissue cancer. Clinicians can access additional information from the full text of papers from the Navigator to make better decisions about patient treatment.

**Table 3:** Outcome statistics for all tissue types.

Tissue type with cancer	Patients (n)	Deaths due to COVID-19 (% fatality), n (%)
Bladder/urinary tract	1	0 (0)
Bone and soft tissue	9	3 (33)
Bowel	5	0 (0)
Brain/central nervous system	2	0 (0)
Breast	91	5 (6)
Cervix	1	0 (0)
Genitourinary	4	0 (0)
Gynecological	180	20 (11)
Head and neck	8	0 (0)
Hematologic not specified	1893	492 (26)
Kidney	18	2 (11)
Liver	5	1 (20)
Lung	225	67 (30)
Lymphoid	504	152 (30)
Myeloid	29	5 (17)
Not specified	13265	2632 (20)
Ovary/fallopian tube	2	0 (0)
Pleura	1	0(0)
Prostate	124	28 (23)
Skin	3	0 (0)
Thoracic	200	66 (33)
Thymus	1	0 (0)

## Discussion

### Views From Clinician Users

We surveyed clinicians who tested the SMART COVID Navigator to elicit their assessment. Their feedback highlighted the following uses of the application in patient care and as an educational resource. The clinicians noted that the application allows for a real-time assessment of comorbidities that any given patient may have that could impact severity and fatality risk from COVID-19 and that it allows for the assessment of patients in which multiple factors may be at play. The clinicians also noted that the application could be used at the point of care, and this application filled an unmet clinical need. They stated that the application made it easier to narrow down the literature and find out the relevant patient management–related answers quickly. They also pointed out that the fatality and severity rates displayed by the CORD-19 data could help them in triaging and stratifying patients in limited resource conditions, allowing them to decide whether aggressive treatment was warranted immediately or not. This would also help them involve palliative care early enough if needed. Finally, they commented that the application would help them in shared decision-making with patients.

Apart from patient care, the clinicians felt that the SMART COVID Navigator could serve as an educational resource for teaching medical students, residents, journal clubs, and perhaps, even in continuing medical education for physicians. Some also felt that the application can help in making institutional guidelines. The clinicians made suggestions for future work related to the SMART COVID Navigator. Real-time use of the application would be enhanced by creating a smartphone interface in addition to the current desktop-based web interface, considering that physicians often perform literature reviews on smartphones. Although the application currently links to the VA and CMS, some clinicians felt that the application would benefit from being linked to other EHRs such as Epic. In fact, the same type of user interface and back-end API (SMART-on-FHIR) can be leveraged and is supported by these as well. Another suggestion was to link the application to a patient’s COVID-19 vaccination records to further improve the physician’s ability to treat the patient.

### Additional Comments and Extensions

SMART COVID Navigator was created in response to a growing need for precision medicine tools to assist doctors dealing with COVID-19–infected patients with risk factors shown to affect COVID-19 progression. The Navigator achieves this by connecting patient medical information—in the form of EHRs—with data sets giving comprehensive information about patient outcomes. We show in this paper how to create a clinical decision support system for physicians to understand how a new disease (COVID-19) interacts with a broad set of patient risk factors through the use of the CORD-19 data set. In addition, we expand 1 existing patient condition, namely, cancer, and provide physicians information on how various types of cancers interact with COVID-19. This can be extended to other conditions such as heart disease since certain chronic conditions appear with a large number of variations in patients.

This application benefits from the creation of research study data sets such as CORD-19 and the Reboot: COVID-Cancer Project, thereby showing the value of such collaborative efforts to collect current research findings in one place. Currently, these data sets do not provide API access; therefore, we have to periodically download these data sets for use in the app. In the future, we recommend that such data sets provide API access to enable real-time updating. We note that in the CORD-19 data set, some disease categories are examined by a limited number of papers; thus, the results may not be meaningful for our understanding of COVID-19’s impact on patients with that risk factor. Similarly, certain cancer tissue types are not well represented in the Reboot: COVID-Cancer Project data set. We expect that as research into COVID-19 progresses further, there will be better representation across disease categories and tissue types. The ability for users to log into 2 FHIR-supported EHR systems is a useful feature of the application. In addition to providing physicians the latest information about patient’s health records, it can also alert users to discrepancies between different databases, giving them an opportunity to correct their health records. This application is not limited to just the 2 EHR databases currently used (VA and CMS); more EHRs can be added to the application as long as they follow the FHIR format, allowing for even more expansion of patient record access.

A possible next step for the SMART COVID Navigator could be to better consolidate the research available in CORD-19 and the Reboot: COVID-Cancer Project by using meta-analysis of the various studies to provide more information to the clinician [24]. For instance, more sophisticated weighting of the results in different papers could be implemented. Another extension would be to provide treatment information through the Navigator platform depending upon the patient’s health profile. In addition, advancements in artificial intelligence and machine learning technologies could play an important role in the improvement of the Navigator platform, as well as precision medicine as a whole, by offering predictions about disease progression based on past patient health information, thereby tailoring treatment for the individual and allowing for increased efficiency in treatment [25]. This could take the form of creating patient risk scores generated based on data from EHRs.

### Conclusion

Precision medicine has long been hailed as the next step in advancing patient care, with institutions such as the White House creating initiatives to further precision medicine technology advancement [26]. The SMART COVID Navigator is a clinical decision support tool designed to allow physicians to provide precision medicine in the context of COVID-19. Using the app, clinicians can identify patient risk factors from multiple EHRs and connect them to databases of COVID-19 research. Without a clinical decision support system supporting COVID-19 precision medicine, a clinician would need to examine multiple studies in order to fully understand disease progression and fatality outcomes, given a particular disease that their patient has, which is costly in terms of time and effort. The number of studies examining particular disease conditions and their relationship to COVID-19 is growing at a rapid pace. This application simplifies this by providing summary statistics on the risk factors’ effect on COVID-19 severity and fatality. Although this application currently focuses on COVID-19, it can be a readily available platform for quickly expanding into any potential new diseases that emerge.

## Abbreviations

API: application programming interface
CMS: Center for Medicare and Medicaid Services
CORD-19: COVID-19 Open Research Dataset
EHR: electronic health record
FHIR: Fast Healthcare Interoperability Resources
ICD-10: International Classification of Diseases-tenth version
VA: Veterans Affairs
